# Cohort Profile: The Fangshan Cohort Study of Cardiovascular Epidemiology in Beijing, China

**DOI:** 10.2188/jea.JE20120230

**Published:** 2014-01-05

**Authors:** Na Wu, Xun Tang, Yiqun Wu, Xueying Qin, Liu He, Jinwei Wang, Na Li, Jingrong Li, Zongxin Zhang, Huidong Dou, Jianjiang Liu, Liping Yu, Haitao Xu, Jianguo Zhang, Yonghua Hu, Hiroyasu Iso

**Affiliations:** 1 Department of Epidemiology and Biostatistics, Peking University Health Science Center, Beijing, China; 2 Fangshan District Bureau of Health, Beijing, China; 3 The First Hospital of Fangshan District, Beijing, China; 4 Public Health, Department of Social and Environmental Medicine, Graduate School of Medicine, Osaka University, Suita, Osaka, Japan

**Keywords:** risk factors, cardiovascular disease, rural population, cohort study, China

## Abstract

**Background:**

Urbanizing rural areas in China face a rapidly growing cardiovascular disease burden. Epidemiologic studies and effective preventive strategies are urgently needed.

**Methods:**

The Fangshan Cohort Study is a prospective study that began in 2008 and targets local residents aged 40 years or older living in 3 towns in the Fangshan district of Beijing. The baseline examination included a questionnaire on medical history, health knowledge, and behaviors related to cardiovascular disease, as well as physical and blood biochemical examinations. The questionnaire survey will be readministered every 2 years. A system for surveillance of mortality and morbidity of cardiovascular disease is under development.

**Results:**

A total of 20 115 adults (6710 men and 13 405 women) were investigated at baseline (participation rate = 84.5%). The data indicate that overweight/obesity is a serious public health issue in Fangshan: average body mass index was 25.4 kg/m^2^ among men and 26.5 kg/m^2^ among women, and the prevalences of overweight and obesity were 43.6% and 10.3% among men and 47.0% and 17.7% among women.

**Conclusions:**

The Fangshan Cohort Study will provide data on cardiovascular risk factors and disease profile, which will assist in developing appropriate prevention and control strategies for cardiovascular disease in rural Chinese communities.

## INTRODUCTION

Cardiovascular disease (CVD) is the leading cause of death in the world and accounted for 23.6% of all deaths in 2008.^1^ Mortality from CVD has declined in high-income countries but is increasing in many developing countries, such as China.^2^ Indeed, China is facing a growing epidemic of CVD.

China is a large agricultural country. In 2006, 737 million people were living in rural communities—56% of the entire Chinese population.^3^ In China, an urban area is defined as a prefecture-level city or larger community and a rural area as a county or smaller community. There are 4 economic categories for rural areas. Annual per capita net income (in renminbi) for rural residents is classified as 3000 RMB or higher, 2000 to 2999 RMB, 1500 to 1999 RMB, and less than 1500 RMB for first- to fourth-class rural areas, respectively.^4^ The income gap between urban and rural areas has widened with the increase in economic development that began in the 2000s. Therefore, many young workers migrate to large and medium-sized cities to seek jobs, leaving elderly people in the countryside. Along with the structural transformation of the economy, lifestyles (including diet and physical activity) have also changed. The traditional diet is made up mainly of cereals and is low in fat and calories and high in carbohydrate and dietary fiber. During the last 20 years, consumption of cereals has decreased rapidly and consumption of animal products has increased,^5^ which may have accelerated the epidemic of CVD.^6^^,^^7^ In some developed rural areas, cardiovascular disease morbidity and mortality exceed levels in urban areas.^8^^–^^10^ However, because of the lack of health awareness, uneven distribution of health resources, long distances to hospitals, and low incomes, rural areas in China may have more challenges in preventing and controlling CVD.

Large-scale cohort studies have examined the secular trend and epidemiologic characteristics of CVD in China.^7^^,^^11^^–^^13^ However, these cohort studies were conducted in the 1990s and ended in around 2000. Thus, data for recent years are lacking, especially for rural populations. In addition to academic research, the Chinese government is addressing the issue of chronic disease in rural areas. For example, the New Rural Cooperative Medical System was established in 2003.^14^ This health care system targets the rural population and is organized, led, and supported by the government, with the voluntary participation of rural residents. The system is jointly financed by individuals, collectives, and the government and attempts to reduce illness-induced poverty and reimburse the cost of major illnesses.^15^ Excepting Hong Kong and Macao, it covers all 22 provinces, 4 municipalities, and 5 autonomous regions in China. A total of 832 million rural residents (96% of the entire rural population of China) were covered by this system as of 2011.^16^ The Chronic Disease Record was started in 2009 and includes demographic information, family history, medical history, outpatient record, and other information for every resident (as recorded by community medical centers). In 2011, it included 30% of rural residents nationwide.^17^ All these policies and programs are important in preventing and controlling chronic diseases in rural areas.

Because the epidemiologic patterns of CVD change quickly in Chinese rural areas, we analyzed (1) CVD trends in rural populations, (2) awareness, treatment, and control of CVD, and their contributing factors, (3) the burden of chronic diseases, and local health needs, (4) medications commonly used for treating chronic diseases and their long-term beneficial effects, adverse effects, compliance, and pharmacoeconomics, and (5) effective preventive and control strategies that were specially developed for rural populations. These data will be useful in devising health policies to address the epidemic of CVD.

## METHODS

The main reason for developing the Fangshan cohort study was to analyze the changing epidemiologic characteristics of CVD among rural populations, including morbidity, mortality, prevalence, and risk factors. To investigate awareness, treatment, and control of CVD, we will conduct repeat surveys of medical history and medication adherence, as measured by the Morisky Scale.^18^ The burden of chronic disease will be measured by potential years of life lost (PYLL),^19^ disability-adjusted life years (DALYs),^20^ and the medical cost of the disease. Our ultimate goal is to collect data that assist in the development of suitable preventive strategies. This will require identification of the risk factor profile and sensitive biomarkers for CVD, and the establishing of intervention priorities, after which effective, economical treatment methods can be specially developed for rural populations.

### Study design, setting, and participants

The prospective study started in 2008, and the targeted population was local rural residents aged 40 years or older living in the Fangshan district of Beijing, 12.5 miles southwest of downtown Beijing (Figure). People were excluded if they had a severe physical or mental disease that made them unable to answer the questionnaire or if they had a severe medical condition that made them unable to report to the survey location. Fangshan occupies an area of 2019 km^2^ and comprises mountains, hills, and plateaus. It includes 8 subdistricts, 14 towns, and 6 townlets (the smallest administrative unit in China, based on the Constitution Law of 1982). The census population was 870 000, the rural population was greater than 400 000, and the population is relatively stable. Fangshan district has a high prevalence of CVD.^21^^–^^23^ Local government and residents are cooperative, and the present authors have been involved in the area for other research projects since 1981.

**Figure.**
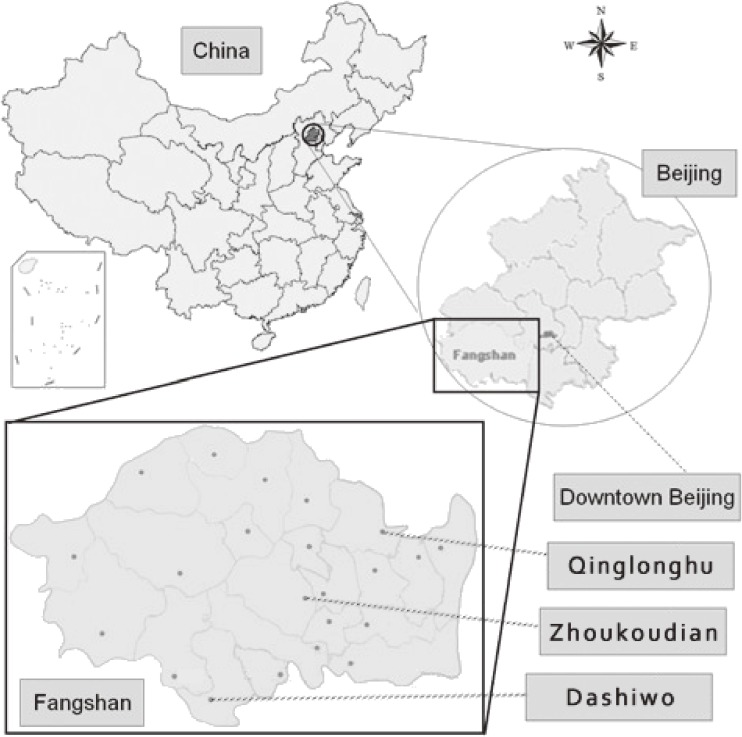
Map showing the locations of the 3 towns studied in the Fangshan Cohort Study.

A stratified, multistage, cluster-sampling design was used in the present study. A random sample of 3 towns (Zhoukoudian, Dashiwo, and Qinglonghu) was selected to represent the 3 different topographical areas (mountain, hill, and plateau, respectively), because both health knowledge and the conditions of the residents differ among these areas. The 3 selected towns are located in the north, center, and south of Fangshan district (in ascending order of distance from downtown Beijing). As in previous preliminary studies, we used inference for a single proportion to calculate sample size^24^ for the 3 towns, to detect regional differences in CVD prevalence.^23^^,^^25^ In Fangshan district the prevalence of stroke was lower than the prevalences of hypertension, coronary heart disease, and diabetes mellitus, according to our preliminary studies.^23^^,^^25^ Using inference for a single proportion to calculate sample size,^24^ the absolute precision was set as 10 percentage points of the expected prevalence, and the confidence level was set as 95%. The expected prevalence of stroke was 4.9%, 4.8%, and 4.3% for people aged 40 years or older in Zhoukoudian, Dashiwo, and Qinglonghu, respectively, according to our pilot study. After calculating the sample size for each town, we used cluster sampling, with the village as the unit. We calculated the proportion of people that had to be sampled from the census population in each town. That proportion was almost equal to the proportion of the village that needed to be sampled from the town, because the census population of the village is nearly identical. After the calculation, 14, 10, and 18 villages needed to be sampled out of the 24, 24, and 20 villages in Zhoukoudian, Dashiwo, and Qinglonghu, respectively. Within towns we used simple random cluster sampling for villages, because there is no heterogeneity of characteristics among different villages in the same town. In 2008, the annual per capita net income for rural residents (US dollars) was $1408 for Zhoukoudian, $1218 for Dashiwo, and $1254 for Qinglonghu. It ranged from $651 to $2446 for other areas and was $1450 for the Fangshan district overall.^26^ Therefore, the economic levels of the 3 towns did not substantially differ from that of the Fangshan district overall and were higher than the national average for rural areas ($686 in 2008).

In 2008, the baseline survey was conducted for the sampling subjects (*n* = 7514) in Zhoukoudian town, which had a census population of 12 674 adults aged 40 years or older; 6047 chose to participate (participation rate = 80.5%). In 2009, the survey was conducted for the sampling subjects (*n* = 7728) in Dashiwo town, which had a census population of 17 872 adults aged 40 years or older; 6211 (80.4%) chose to participate. In 2010, the survey was conducted for the sampling subjects (*n* = 8571) in Qinglonghu town, which had a census population of 9753 adults aged 40 years or older; 7857 (91.7%) chose to participate. Thus, a total of 20 115 adults participated in the baseline survey of the 3 towns, and the overall participation rate was 84.5%.

The baseline survey included an interview and physical and blood biochemical examinations and was conducted at the community medical center of each sampled village. To recruit participants, the staffs of the local village governments publicized the survey through broadcasts and household telephone 1 day before and during the survey. The interview was done by trained investigators using a uniform questionnaire, the physical examination was conducted by research physicians and nurses, and the blood samples were processed by laboratory technicians.

### Baseline measures

The main measures of the baseline examination are summarized in Table 1. Additional information for some items was investigated in the 2010 questionnaire survey.

**Table 1.** Summary of the baseline measures in Fangshan Cohort Study

Demographic information	
	Age
	Sex
	Ethnic group
	Marital status
	Education background
	Occupation
	Annual income
Medical history	
	Hypertension, diabetes mellitus, coronary heart disease, stroke
	Family history of hypertension, diabetes mellitus, coronary heart disease, stroke
Health knowledge and behaviors	
	Willingness and methods to obtain health information
	Smoking status
	Alcohol consumption
	Physical exercise (frequency of exercise, type of exercise)
	Dietary pattern (preference for tea, meat, oil, sweet food, salty food)
	Sleep duration
Quality of life	
	Assessed by self-rated health with 5 rating levels, or the EQ-5D scale^a^
Demand and utilization of health service^a^	
Physical examination	
	(Resting blood pressure, height, weight, waist and hip circumferences,^a^ 12-lead resting electrocardiogram)
Blood biochemical examination	
	Total cholesterol, triglycerides, HDL-cholesterol, LDL-cholesterol, blood glucose

Abbreviations: EQ-5D, European Quality of Life–5 Dimensions scale; LDL, low-density lipoprotein; HDL, high-density lipoprotein.

^a^Additional information investigated in 2010 questionnaire survey.

The baseline questionnaire included the following individual-level information: demographic factors (age, sex, marriage status, education level, and occupation), medical history (year of diagnosis and medication used for hypertension, diabetes mellitus, coronary heart disease, and stroke diagnosed by a class 2 or higher hospital). To investigate genetic epidemiology, we also obtained a detailed family history of CVD so that we could collect information on pedigrees, sib-pairs, and twins. Investigated lifestyle factors included smoking status and number of cigarettes smoked per day (current smokers were defined as people who had smoked more than 100 cigarettes in the past and had smoked during the previous 30 days; ex-smokers were defined as people who had smoked more than 100 cigarettes in the past but had not smoked during the previous 30 days), drinking status (current drinkers were defined as persons who reported current consumption of alcohol at least once a week; ex-drinkers were defined as people who reported consuming alcohol at least once a week in the past but not during the previous 30 days), regular physical exercise (defined as intentional exercise for at least 30 minutes at least once per week during the previous 6 months, not including housework or job-related work), dietary pattern (preference for tea, meat, oil, sweet food, or salty food), and hours of sleep per night. The definition of smoking was the same as that used in the Chinese National Health Services Survey in 2008,^4^ and the definitions for drinking and regular physical exercise were the same as those used in the Chinese National Health Services Survey in 2008 and National Nutrition and Health Survey in 2002.^4^^,^^27^ Regular physical exercise was determined by asking the question, “Do you intentionally exercise?”, and the responses “always” (for at least 30 minutes ≥3 times per week) and “sometimes” (at least 30 minutes once or twice per week) were regarded as an affirmative response. Quality of life was assessed by the European Quality of Life–5 Dimensions (EQ-5D) scale^28^ in 2010, and by self-rated health, with 5 rating levels, before that. Participant knowledge of CVD, and willingness and common approaches to obtain such knowledge, were also surveyed. We asked if they understood the relations between lifestyle and traditional risk factors and between risk factors and CVD. Further, we ascertained their willingness to obtain more information on healthy living, the media they most frequently consulted, and frequency of watching TV programs on the Fangshan Health Channel. We used some of the questions from the Chinese National Health Services Survey to measure resident health-service demands, utilization, and expenditure. Because the New Rural Cooperative Medical System was established in 2003, we also asked about participant satisfaction and comments regarding the system.

The physical examination comprised resting blood pressure, height, weight, waist circumference, hip circumference, and a 12-lead resting electrocardiogram (ECG). Systolic and fifth-phase diastolic blood pressures in the right arm were measured 3 times by trained physicians using standard mercury sphygmomanometers and a standard epidemiologic method. The participants were asked to sit and rest for 5 minutes before measurements.^29^ Hypertension was defined as an average systolic blood pressure of 140 mm Hg or higher, an average diastolic blood pressure of 90 mm Hg or higher, and/or use of antihypertensive medications, according to the 1999 World Health Organization International Society of Hypertension Guidelines.^30^ Diabetes mellitus was defined as a fasting glucose level of 7.0 mmol/l or higher, a random glucose level of 11.1 mmol/l or higher, and/or use of insulin or oral hypoglycemic agents, according to the 1999 World Health Organization Guidelines.^31^ Height was measured by using a fixed stadiometer. Participants were asked to remove their shoes and hats, stand with heels, hips, and shoulders to the wall, look straight ahead, and keep their shoulders horizontal. The measurements were accurate to 1 cm. Weight was measured by a calibrated weighing scale. Participants were asked to remove heavy clothes and shoes. Measurements were accurate to 1 kg. Waist and hip circumferences were measured while the participant was standing. Waist circumference was measured with the tape at the midpoint between the lower costal margin and the iliac crest; hip circumference was measured at the level of maximal extension of the buttocks. The 12-lead resting ECG was measured by a standard method.^32^

Venous blood samples were sent to the laboratory of The First Hospital of Fangshan District for measurement of total cholesterol, triglycerides, high-density lipoprotein cholesterol, low-density lipoprotein cholesterol (measured directly), and blood glucose. Serum was used for the assay, and blood samples were stored at −20°C for DNA extraction. The lipids and glucose were analyzed by a Hitachi 7060 Automatic Biochemical Analyzer (Hitachi High-Technologies Corp., Tokyo, Japan). All samples were assayed in the same laboratory with the same analyzer. Quality control of the laboratory was maintained by internal standardization. In addition, the laboratory of the First Hospital of Fangshan District is under external standardization with the Beijing Central Clinical Laboratory.

### Follow-up and outcome measures

Follow-up surveys have been conducted every 2 years since 2010. The information collected is the same as that obtained for the baseline survey, except for family history. We also gathered information on outpatient records, to evaluate CVD treatment and control. This information was collected from community medical centers (which are present in every village). A morbidity and mortality surveillance system is currently under development. Fangshan joined the WHO-MONICA project in 1984,^33^ and a registry system has been in place since then. The surveillance system is based on that registry system. The process and criteria for case ascertainment and validation are identical to those used in the WHO-MONICA project.^34^ The surveillance system comprises the community medical centers, 3 township hospitals (each town has 1 township hospital), and 3 upper-class-2 hospitals (Liangxiang Hospital, the First Hospital of Fangshan District, and Fangshan Traditional Chinese Medicine Hospital).

### Ethical issues

The study protocol and informed consent procedure were approved by the Ethics Committees of Peking University Health Science Center. All study participants signed the informed consent form before taking part in the survey.

### Statistical analysis

Age- and sex-specific descriptive statistics are presented as mean (SD) for continuous variables and as frequency counts and proportions for categorical data. To test differences in means and proportions among the 3 towns, we used analysis of variance and the χ^2^ test, respectively. The Statistical Program for Social Sciences, Version 17.0 (SPSS Inc., Chicago, IL) was used for all statistical analyses. A 2-tailed *P* value of less than 0.05 was considered to indicate statistical significance.

## RESULTS

Table 2 summaries the baseline characteristics of the participants. Mean age was 56.6 years for men and 55.6 years for women; 90.6% of men and 86.3% of women were married, and 7.0% of men and 12.8% of women were widowed. The proportion of married respondents decreased, and the proportion of widowed participants increased, with increasing age. The proportion of single/divorced participants was 0.3% to 1.4% for both sexes. The most commonly reported level of education was junior high school (53.1% of men and 38.3% of women). A primary school education was the second most frequent level of education (22.8% of men and 28.2% of women). Few people had entered university (only 0.1% for both sexes). As age increased, the proportion of people who had never attended school increased and the proportions of those with a junior high school or high school education decreased.

**Table 2.** Baseline characteristics of the Fangshan Cohort Study

	Men						Women					
	
Age, years	40–49	50–59	60–69	70–79	≥80	Total	40–49	50–59	60–69	70–79	≥80	Total
Number	1917	2463	1483	726	121	6710	4134	5118	2797	1172	184	13 405

Marital status (%)												
Single	1.2	1.7	1.3	1.3	0.9	1.4	0.2	0.2	0.6	0.5	0.5	0.3
Married	96.3	94.0	90.0	71.2	52.2	90.6	97.4	93.4	77.5	47.1	25.5	86.3
Divorced	1.5	1.1	0.6	0.4	0	1.0	0.7	0.6	0.3	0.4	0.5	0.5
Widowed	1.0	3.1	8.1	27.0	47.0	7.0	1.7	5.8	21.7	52.1	72.6	12.8

Education (%)												
Never	2.2	6.5	12.4	39.4	60.0	11.1	3.0	20.1	34.2	73.1	86.8	23.4
Primary school	7.5	21.8	31.9	47.2	26.1	22.8	12.2	35.8	41.7	22.5	10.4	28.2
Junior high school	65.4	59.2	50.8	11.8	10.4	53.1	64.8	34.7	23.0	3.5	2.7	38.3
High school	24.9	12.4	4.8	1.4	3.5	12.9	20.0	9.4	1.1	0.8	0	10.0
University	0	0	0.1	0.1	0	0.1	0.1	0	0	0	0	0.1

Smoking status (%)												
Never	26.4	25.6	27.6	29.6	36.8	26.9	92.5	89.3	77.7	68.0	75.0	85.8
Ex-smoker	9.5	15.2	19.7	26.0	28.9	15.9	0.7	1.9	5.1	10.6	10.3	3.1
Current	64.0	59.2	52.7	44.4	34.2	57.1	6.8	8.8	17.1	21.4	14.7	11.1

Drinking status (%)												
Never	37.5	38.4	38.4	43.3	44.7	38.7	93.5	93.8	92.1	88.7	88.5	92.8
Ex-drinker	6.9	11.4	12.8	18.7	18.4	11.3	0.5	0.8	1.6	3.5	1.6	1.1
Current	55.5	50.2	48.8	38.0	36.8	49.9	5.9	5.5	6.3	7.8	9.8	6.1

Regular physical exercise (%)^a^												
No	71.0	64.8	54.6	49.9	51.8	62.6	62.4	57.7	47.8	47.5	62.7	56.3
Yes	29.0	35.2	45.4	50.1	48.2	37.4	37.6	42.3	52.2	52.5	37.3	43.7

Taste preference (%)												
Salty	45.2	44.7	40.9	33.9	35.4	42.6	37.1	35.6	31.6	27.7	18.5	34.3
Somewhat salty	36.7	36.8	34.3	40.4	27.4	36.5	39.5	39.8	40.7	42.5	46.7	40.2
Not salty	18.1	18.4	24.9	25.7	37.2	20.9	23.5	24.7	27.7	29.7	34.8	25.5

Prevalence (%)												
Hypertension^b^	58.7	63.9	68.5	73.3	69.3	64.5	51.6	61.5	70.3	76.8	73.1	61.8
Diabetes mellitus^c^	10.8	13.3	12.2	12.1	8.3	12.1	8.4	14.5	19.1	14.8	14.2	13.6

Medical history (%)												
Hypertension	34.2	41.9	53.0	55.6	49.1	43.8	33.4	47.9	57.7	64.2	61.2	47.2
Diabetes mellitus	10.6	13.2	11.8	11.3	7.8	11.9	8.6	15.5	20.7	15.5	15.3	14.5
Coronary heart disease	7.5	12.6	21.3	24.9	17.1	14.5	8.2	18.0	30.3	37.5	37.2	19.6
Stroke	5.0	13.0	20.9	23.5	16.4	13.7	2.7	7.6	14.2	15.5	14.2	8.3

BMI (kg/m^2^)	26.2 ± 3.6	25.5 ± 3.4	24.9 ± 3.4	24.1 ± 3.7	23.8 ± 3.8	25.4 ± 3.6	26.6 ± 3.7	26.8 ± 3.8	26.4 ± 4.0	25.4 ± 4.2	24.4 ± 3.9	26.5 ± 3.9

BMI (%)												
<18.5	0.7	1.2	2.3	4.1	5.8	1.7	0.3	0.6	1.5	3.9	5.0	1.1
18.5–24.9	35.7	42.9	50.4	57.9	61.7	44.5	34.3	30.9	35.7	42.5	50.9	34.2
25–29.9	49.6	45.6	39.9	31.5	26.7	43.6	48.0	49.3	44.9	39.4	37.9	47.0
≥30	14.1	10.4	7.4	6.6	5.8	10.3	17.3	19.1	17.9	14.3	6.2	17.7

Abbreviation: BMI, body mass index.

^a^Defined as intentional exercise for ≥30 minutes and at least once per week during previous 6 months, not including housework or job-related work.

^b^Defined as average systolic blood pressure ≥140 mm Hg, average diastolic blood pressure ≥90 mm Hg, and/or use of antihypertensive medications.

^c^Defined as fasting glucose ≥ 7.0 mmol/l, random glucose ≥ 11.1 mmol/l, and/or use of insulin or oral hypoglycemic agents.

Regarding smoking status, 57.1% of men and 11.1% of women were current smokers, and 26.9% of men and 85.8% of women were never smokers. As age increased, the proportion of current smokers decreased among men and increased among women.

As for alcohol consumption, 49.9% of men and 6.1% of women were current drinkers, and 38.7% of men and 92.8% of women were never drinkers. As age increased, the proportion of current drinkers decreased among men and increased among women, as was the case for smoking. Overall, 37.4% of men and 43.7% of women reported engaging in regular physical exercise. Excepting adults aged 80 years or older, the proportion of adults taking part in regular physical exercise increased with age.

Regarding dietary preference, 42.6% of men and 34.3% of women reported preferring salty foods. This preference was more prevalent among younger age groups in both sexes. The prevalence of hypertension was 64.5% among men and 61.8% among women. The prevalence of diabetes mellitus was 12.1% among men and 13.6% among women. The proportions of respondents who reported a diagnosis of hypertension, diabetes mellitus, coronary heart disease, and stroke were 43.8%, 11.9%, 14.5%, and 13.7%, respectively, among men and 47.2%, 14.5%, 19.6%, and 8.3%, respectively, among women.

Mean BMI was 25.4 kg/m^2^ among men and 26.5 kg/m^2^ among women; 43.6% of men and 47.0% of women were overweight (BMI 25.0–29.9 kg/m^2^) and 10.3% of men and 17.7% of women were obese (BMI ≥30.0 kg/m^2^). Mean BMI and the proportions of overweight and obesity were higher among younger as compared with older age groups.

All characteristics of the participants from the 3 towns significantly differed, except for prevalence of diabetes mellitus among men (Table 3). The men and women in Qinglonghu had higher educational levels, and lower proportions of current drinkers, as compared with participants in the other 2 towns. The men and women in Dashiwo had the lowest educational level and the lowest proportion of regular physical exercise.

**Table 3.** Baseline characteristics by town (*n*, [%])

	Men			*P* for difference	Women			*P* for difference
	
	Zhoukoudian	Dashiwo	Qinglonghu		Zhoukoudian	Dashiwo	Qinglonghu	
Age (mean ± SD)	55.6 ± 10.0	57.6 ± 17.8	56.5 ± 10.0	<0.001	55.4 ± 10.0	55.6 ± 9.8	55.8 ± 9.6	0.110

Age groups				<0.001				<0.001
40–49	618 (31.8)	520 (26.1)	779 (28.1)		1305 (31.8)	1304 (30.9)	1525 (30.0)	
50–59	736 (37.8)	706 (35.4)	1021 (36.9)		1597 (38.9)	1599 (37.9)	1922 (37.8)	
60–69	376 (19.3)	484 (24.2)	623 (22.5)		746 (18.2)	888 (21.1)	1163 (22.9)	
70–79	184 (9.5)	249 (12.5)	293 (10.6)		406 (9.9)	365 (8.7)	401 (7.9)	
≥80	32 (1.6)	37 (1.9)	52 (1.9)		47 (1.1)	59 (1.4)	78 (1.5)	

Marital status				<0.001				0.001
Single	12 (0.7)	42 (2.1)	37 (1.4)		18 (0.5)	5 (0.1)	17 (0.3)	
Married	1543 (92.5)	1827 (89.6)	2459 (90.1)		2985 (85.0)	3747 (87.5)	4373 (86.2)	
Divorced	16 (1.0)	12 (0.6)	38 (1.4)		16 (0.5)	17 (0.4)	36 (0.7)	
Widowed	96 (5.8)	157 (7.7)	195 (7.1)		494 (14.1)	513 (12.0)	645 (12.7)	

Education				<0.001				<0.001
Never	172 (10.3)	380 (18.6)	160 (5.9)		783 (22.3)	1502 (35.1)	731 (14.4)	
Primary school	358 (21.4)	527 (25.9)	583 (21.4)		1019 (29.0)	1110 (25.9)	1504 (29.7)	
Junior high school	885 (53.0)	937 (46.0)	1597 (58.6)		1368 (38.9)	1362 (31.8)	2195 (43.3)	
High school	244 (14.6)	189 (9.3)	382 (14.0)		346 (9.8)	304 (7.1)	635 (12.6)	
University	12 (0.7)	5 (0.2)	4 (0.1)		2 (0.1)	4 (0.0)	1 (0.0)	

Smoking status				0.003				<0.001
Never	500 (29.6)	507 (24.9)	725 (26.8)		2920 (82.3)	3756 (87.7)	4367 (86.6)	
Ex-smoker	288 (17.1)	323 (15.8)	414 (15.3)		156 (4.4)	97 (2.3)	144 (2.9)	
Current	900 (53.3)	1208 (59.3)	1562 (57.8)		470 (13.3)	431 (10.1)	531 (10.5)	

Drinking status				<0.001				<0.001
Never	689 (40.8)	573 (28.1)	1229 (45.5)		3205 (90.2)	3927 (91.7)	4822 (95.6)	
Ex-drinker	185 (11.0)	289 (14.2)	253 (9.4)		66 (1.9)	51 (1.2)	29 (0.6)	
Current	815 (48.3)	1176 (57.7)	1220 (45.2)		281 (7.9)	306 (7.1)	193 (3.8)	

Regular physical exercise^a^				<0.001				<0.001
No	743 (45.1)	1635 (80.2)	1636 (59.9)		1347 (38.7)	3148 (73.5)	2741 (54.0)	
Yes	905 (54.9)	403 (19.8)	1094 (40.1)		2133 (61.3)	1135 (26.5)	2338 (46.0)	

Taste preference				<0.001				<0.001
Salty	716 (42.5)	751 (36.8)	1270 (47.1)		1213 (34.3)	1303 (30.4)	1894 (37.6)	
Somewhat salty	610 (36.2)	839 (41.2)	892 (33.1)		1398 (39.5)	1823 (42.6)	1943 (38.6)	
Not salty	358 (21.3)	448 (22.0)	536 (19.9)		929 (26.2)	1158 (27.0)	1197 (23.8)	

Prevalence								
Hypertension^b^	1362 (78.5)	1539 (67.5)	1427 (53.0)	<0.001	2561 (72.7)	3258 (65.6)	2465 (49.9)	<0.001
Diabetes mellitus^c^	224 (12.9)	251 (11.0)	331 (12.3)	0.155	451 (12.8)	621 (12.5)	736 (14.9)	0.001

Medical history								
Hypertension	864 (50.0)	852 (42.7)	1104 (40.7)	<0.001	1787 (49.5)	2004 (47.5)	2285 (45.3)	<0.001
Diabetes mellitus	236 (13.6)	187 (9.4)	339 (12.6)	<0.001	499 (13.8)	573 (13.6)	788 (15.7)	<0.001
Coronary heart disease	279 (16.1)	331 (16.6)	320 (11.9)	<0.001	757 (21.0)	812 (19.3)	957 (19.0)	<0.001
Stroke	154 (8.9)	315 (15.8)	411 (15.2)	<0.001	192 (5.3)	369 (8.8)	504 (10.0)	<0.001

BMI	25.7 ± 3.3	25.2 ± 3.7	25.4 ± 3.6	<0.001	26.5 ± 3.8	26.8 ± 3.8	26.2 ± 3.9	<0.001

BMI group				<0.001				<0.001
<18.5	22 (1.4)	36 (1.5)	55 (2.1)		37 (1.0)	39 (0.8)	63 (1.3)	
18.5–24.9	631 (38.9)	1172 (49.0)	1168 (43.8)		1201 (33.8)	1466 (31.5)	1811 (37.0)	
25–29.9	805 (49.6)	946 (39.5)	1160 (43.5)		1685 (47.5)	2232 (47.9)	2238 (45.8)	
≥30	166 (10.2)	240 (10.0)	281 (10.5)		626 (17.6)	919 (19.7)	776 (15.9)	

Abbreviation: BMI, body mass index.

^a^Defined as intentional exercise for ≥30 minutes and at least once per week during previous 6 months, not including housework or job-related work.

^b^Defined as average systolic blood pressure ≥140 mm Hg, average diastolic blood pressure ≥90 mm Hg, and/or use of antihypertensive medications.

^c^Defined as fasting glucose ≥7.0 mmol/l, random glucose ≥11.1 mmol/l, and/or use of insulin or oral hypoglycemic agent.

## DISCUSSION

Fangshan is a rapidly urbanizing rural area and thus provides a good setting to investigate changes in cardiovascular risk factors and disease among rural populations in such conditions. Previous large cohort studies include the China Multi-provincial Cohort Study of 28 594 residents in 11 provinces (started in 1992; 12-year follow-up),^11^ a prospective study of 5137 male steel workers in Beijing (21-year follow-up),^13^ the USA-PRC collaborative study of 11 336 men and women in Beijing and Guangzhou (17-year follow-up),^12^ and the Sino-MONICA project investigation of 5 million people in 16 provinces (7-year follow-up), which monitored trends and determinants of CVD.^7^ However, all these studies ended around the year 2000. The Fangshan Cohort Study will provide data on current cardiovascular epidemiology.

A limitation of the Fangshan Cohort study is the representativeness of the Chinese rural population. China is a large country, with great diversity among its different regions. There may be large differences in CVD incidence, prevalence, and mortality among different rural areas.^35^ Nevertheless, the Fangshan district is representative of developed rural areas in northern China. Because the examinations were free, and due to the good relationship between the city government and our university, the participation rate was high. Therefore, potential bias due to nonparticipation is unlikely to be a concern.

Regarding resident characteristics, most participants were married. In addition 10% to 13% of participants had been in high school or university, which was lower than the 2010 national level in the same age group (17.1%).^36^ The proportion of current smokers was 57.1% among men and 11.1% among women, which was similar to the 2008 national prevalence (about 58%) among men aged 40 years or older but higher than the national prevalence (3%) among women.^37^ Fangshan had a higher prevalence of smoking than did rural communities in Japan (53% among men and 3% among women aged 40 to 69 years in 2003^38^). Unlike the trend toward higher smoking prevalence with increasing age among women in China,^39^ prevalence was higher among young women than among elderly women in Japan. Also, among Japanese women aged 20 to 29 years, the prevalence of smoking increased from 7% in 1965 to 15% in 2008.^40^ In contrast, among Chinese women in the same age group the prevalence of smoking decreased slightly from 1.2% in 1996 to 1.0% in 2002.^41^

Overall, 49.9% of men and 6.1% of women in our survey were current drinkers. As compared with 2002 national levels (43.3% and 6.8%, respectively, for age ≥40 years^42^), the prevalence was higher for men and similar for women.

Overweight/obesity appears to be a serious public health issue in Fangshan. Average BMI was 25.4 kg/m^2^ among men and 26.5 kg/m^2^ among women, and the prevalence of overweight/obesity was 53.3% among men and 64.7% among women. These values are much higher than the 2002 national levels (25.2% and 29.2% for age ≥45 years).^43^ The prevalences of overweight/obesity in the rural districts of Beijing have been among the nation’s highest since the 1980s.^44^ Differences between rural areas of Beijing and other provinces in the prevalence of overweight/obesity have not diminished.^44^ Overweight and obesity have traditionally been uncommon among Asians such as the Chinese and Japanese. However, the prevalence of overweight/obesity was much higher in Fangshan than in a rural area of Japan, which had an average BMI of 24.0 kg/m^2^ among men and 24.3 kg/m^2^ among women aged 40 years or older.^38^ According to the 2009 National Health and Nutrition Survey in Japan, among adults aged 40 years or older the proportions of overweight and obesity were 27.6% and 3.4%, respectively, among men and 19.5% and 3.8% among women.^45^ The 2008 NHANES in the United States showed that median BMI was 28.3 kg/m^2^ among men and 27.7 kg/m^2^ among women aged 40 years or older. Among adults aged 40 years or older the prevalences of overweight and obesity were 42.3% and 35.8%, respectively, among men and 31.7% and 35.8% among women.^46^ Average BMI and the prevalence of overweight/obesity among Fangshan residents were between national levels in Japan and the United States.^45^^,^^46^

The high prevalence of overweight/obesity in Fangshan is likely mainly due to decreased activity thermogenesis. According to nutrition surveys done in Fangshan, mean daily energy intake changed from 2368 kcal in 1983 to 2576 kcal in 1992 to 2370 kcal in 1999, which indicates the lack of a marked change over time.^47^^,^^48^ As for exercise activity, 37.4% of men and 43.7% of women in Fangshan reported taking part in regular physical exercise, similar to 2002 national prevalences for the same age group.^27^ Therefore, a decrease in non-exercise activity, along with rapid economic development, may have contributed to the high prevalence of overweight/obesity in Fangshan. In a previous survey of 885 men and 646 women in rural Beijing, 60.0% of men and 52.8% of women reported that their occupational physical activity had decreased during the past 10 years.^49^ Private business owners were most likely to report decreased occupational physical activity, probably because 27.1% of them had previously been peasants and 35.3% had been manual workers in national factories. Moreover, 52.6% of manual workers who did not change jobs reported decreased occupational physical activity, due to mechanization.^49^ Although no research has studied leisure-time non-exercise activity in rural Beijing, the proportion of Chinese households with TV sets increased from 65% in 1989 to 91% in 1997,^50^ and the increase was more evident in rural areas.^51^

The prevalence of hypertension was 64.5% among men and 61.8% among women in our study. These prevalences were much higher than those in the national data and other areas in China, using the same definition as that in the present study.^52^^,^^53^ The InterASIA study of a nationally representative sample, conducted in 2000–2001, reported that hypertension prevalence was 28.6% among men and 25.8% among women aged 35 years or older.^52^ Hypertension prevalence was 35.3% among men and 32.7% among women aged 35 years or older in rural areas of Shanghai in 2004.^53^

The prevalence of diabetes mellitus was 12.1% among men and 13.6% among women in Fangshan, which were also higher than national levels (ie, 4.6% among men and 5.4% among women aged 35–74 years in all rural areas of China in 2001, using the present definition^54^).

In Fangshan, 14.5% of men and 19.6% of women aged 40 years or older reported that they had received a diagnosis of coronary heart disease, and 13.7% of men and 8.3% of women had received a diagnosis of stroke. These percentages were much higher than national levels (1.0% of men and 0.7% of women aged ≥35 years in rural populations of Linyi city for coronary heart disease,^55^ 1.3% of men and 0.8% of women aged ≥40 years for stroke nationally^56^). Our findings are consistent with previous results, which showed that Beijing has had the highest incidence and prevalence of coronary heart disease and stroke since 1987.^56^^,^^57^ In 1987, the incidence of coronary heart disease in Beijing was 70.3/100 000 among men and 31.3/100 000 among women. Anhui and Sichuan provinces had the lowest incidence: 3.3/100 000 among men in Anhui province and 1.3/100 000 among women in Sichuan province.^57^ The prevalence of stroke in Beijing was 6.8% in 1991, while the lowest prevalence (1.2%) was in Henan province.^56^ It is possible that Fangshan residents were more likely to report a medical history of coronary heart disease and stroke because, under the conditions of a policy established in Fangshan in 2008, they could receive free medications once diagnosed. Our surveillance of CVD incidence is expected to reveal a clearer picture of CVD in Fangshan.

In conclusion, the Fangshan Cohort Study will collect useful epidemiologic data on cardiovascular risk factors and disease profile, which will aid in the development of appropriate CVD prevention and control strategies for rural areas of China.

## References

[1] The Top 10 Causevs of Death [Internet]. Geneva: World Health Organization [cited 2011 Aug 23]. Available from: http://www.who.int/mediacentre/factsheets/fs310/en/index.html

[2] Yang G, Kong L, Zhao W, Wan X, Zhai Y, Chen LC, Emergence of chronic non-communicable diseases in China. Lancet. 2008;372:1697–705 10.1016/S0140-6736(08)61366-5 18930526

[3] Data of the Sample Survey in 2005 [Internet]. Beijing: National Bureau of Statistics of China [cited 2011 Aug 25]. Available from: http://www.stats.gov.cn/tjsj/ndsj/renkou/2005/renkou.htm

[4] Report of the third Chinese National Health Services Survey [Internet]. Beijing: Ministry of Health [cited 2012 Mar 19]. Available from: http://www.moh.gov.cn/publicfiles/business/htmlfiles/mohwsbwstjxxzx/s8560/201009/49162.htm

[5] Li LM, Rao KQ, Kong LZ, Yao CH, Xiang HD, Zhai FY, ; Technical Working Group of China National Nutrition and Health Survey [A description on the Chinese national nutrition and health survey in 2002]. Zhonghua Liu Xing Bing Xue Za Zhi. 2005;26(7):478–84 16334996

[6] Liu L Cardiovascular diseases in China. Biochem Cell Biol. 2007;85:157–63 10.1139/O07-004 17534394

[7] Wu Z, Yao C, Zhao D, Wu G, Wang W, Liu J, Sino-MONICA project: a collaborative study on trends and determinants in cardiovascular diseases in China, Part i: morbidity and mortality monitoring. Circulation. 2001;103:462–8 10.1161/01.CIR.103.3.462 11157701

[8] Liu S, Zhao D, Wang W, Liu J, Qin LP, Zeng ZC, The trends of cardiovascular risk factors in urban and rural areas of Beijing during 1984–1999. J Cardiovasc Pulm Dis. 2006;25:129–34 (in Chinese)

[9] Wu G, Wu Z, Liu J [Trend of changes in mortality of cardiovascular diseases in some areas of Beijing during 1984 to 1998]. Zhonghua Yu Fang Yi Xue Za Zhi. 2001;35(2):98–101 11413693

[10] Liu J, Zhao D, Wang W, Sun JY, Li Y, Jia YN [Trends regarding the incidence of recurrent stroke events in Beijing]. Zhonghua Liu Xing Bing Xue Za Zhi. 2007;28(5):437–40 17877169

[11] Liu J, Hong Y, D’Agostino RB Sr, Wu Z, Wang W, Sun J, Predictive value for the Chinese population of the Framingham CHD risk assessment tool compared with the Chinese Multi-Provincial Cohort Study. JAMA. 2004;291:2591–9 10.1001/jama.291.21.2591 15173150

[12] An epidemiological study of cardiovascular and cardiopulmonary disease risk factors in four populations in the People’s Republic of China. Baseline report from the P.R.C.-U.S.A. Collaborative Study. People’s Republic of China–United States Cardiovascular and Cardiopulmonary Epidemiology Research Group. Circulation. 1992;85(3):1083–96 10.1161/01.CIR.85.3.1083 1537106

[13] Zhang XF, Attia J, D’Este C, Yu XH Prevalence and magnitude of classical risk factors for stroke in a cohort of 5092 Chinese steelworkers over 13.5 years of follow-up. Stroke. 2004;35:1052–6 10.1161/01.STR.0000125305.12859.ff 15073407

[14] Wagstaff A, Lindelow M, Jun G, Ling X, Juncheng Q Extending health insurance to the rural population: an impact evaluation of China’s new cooperative medical scheme. J Health Econ. 2009;28:1–19 10.1016/j.jhealeco.2008.10.007 19058865

[15] Klotzbücher S, Lässig P Transformative State Capacity in Post-Collective China: The Introduction of the New Rural Cooperative Medical System in Two Counties of Western China, 2006–2008. Eur J East Asian Stud. 2009;8(1):61–89 10.1163/156805809X439895 21984878 PMC3188853

[16] Report of the Press Conference of Ministry of Health [Internet]. Beijing: Ministry of Health of China [cited 2011 Sep 3]. Available from: http://www.moh.gov.cn/sofpro/cms/previewjspfile/wsb/cms_0000000000000000207_tpl.jsp?requestCode=51995&CategoryID=540

[17] Circular on the Main Tasks of Health System Reform [Internet]. Beijing: State Council of China [cited 2011 Aug 23]. Available from: http://www.gov.cn/zwgk/2009-04/07/content_1279256.htm

[18] Morisky DE, Malotte CK, Choi P, Davidson P, Rigler S, Sugland B, A patient education program to improve adherence rates with antituberculosis drug regimens. Health Educ Q. 1990;17:253–67 10.1177/109019819001700303 2228629

[19] Gardner JW, Sanborn JS Years of potential life lost (YPLL)—what does it measure? Epidemiology. 1990;1:322–9 10.1097/00001648-199007000-00012 2083312

[20] Murray CJ Quantifying the burden of disease: the technical basis for disability-adjusted life years. Bull World Health Organ. 1994;72:429–45 8062401 PMC2486718

[21] Huang GY, Gu DF, Duan XF, Xu XS, Gan WQ, Chen JC, [Effects of 8 years community intervention on risk factors of cardiovascular diseases in Fangshan Beijing]. Zhongguo Yi Xue Ke Xue Yuan Xue Bao. 2001;23(1):15–8 12905810

[22] Li J, Cao W, Hu Y, Zhan S, Li P, Li X, [Evaluation on the effects of community-based comprehensive prevention and control of hypertension in the rural areas in China]. Zhonghua Liu Xing Bing Xue Za Zhi. 2000;21(3):185–9 11860781

[23] Zhao QS, Zhang Q, Wang HJ Epidemiology of hypertension in Fangshan of Beijing. Chin J Prev Contr Chron Non-commun Dis. 2007;15:43–4 (in Chinese)

[24] Lwanga SK, Lesmeshow S. Sample size determination in health studies: a practical manual. Geneva: World Health Organization; 1991. p. 1–2.

[25] Guo YM, Zhang FQ, Gao CS, Yan HY Health condition and health needs of residents in Fangshan district. Chin J Rural Med Pharm. 2008;6:67–8 (in Chinese)

[26] Bureau of Statistics of Fangshan. Fangshan District Statistical Yearbook 2008. 1st ed. Beijing: Fangzhi; 2008.

[27] Wang LD. Report of Chinese National Nutrition and Health Survey in 2002. 1st ed. Beijing: People’s Medical Publishing House; 2005.

[28] Szende A, Oppe M, Devlin N. EQ-5D Value Sets: Inventory, Comparative Review and User Guide. 1st ed. Berlin: Springer; 2007.

[29] Wang JW, He L, Tang X, Qin XY, Chen Q, Liu JJ, A cross sectional study of diabetes, hypertension and related cardiovascular complications in people aged 40 years old and above of rural in Fangshan district, Beijing City. Chin J Dis Contr Prev. 2010;14:590–3 (in Chinese)

[30] 1999 World Health Organization-International Society of Hypertension Guidelines for the Management of Hypertension. Guidelines Subcommittee. J Hypertens. 1999;17(2):151–83 10067786

[31] Alberti KG, Zimmet PZ Definition, diagnosis and classification of diabetes mellitus and its complications. Part 1: diagnosis and classification of diabetes mellitus provisional report of a WHO consultation. Diabet Med. 1998;15:539–53 10.1002/(SICI)1096-9136(199807)15:7<539::AID-DIA668>3.0.CO;2-S 9686693

[32] Tang X, He L, Cao Y, Wang JW, Li N, Tian J, [Gender-specific differences in relative effects of cardiovascular risk factors among rural population]. Beijing Da Xue Xue Bao. 2011;43(3):379–85 21681268

[33] Wang W, Zhao D, Wu ZS, Yao L, Zhou MR Comparison of the trends of incidence rate and mortality rate of acute coronary events between urban and rural area in Beijing. J Cardiovasc Pulm Dis. 1995;14:2–14 (in Chinese)

[34] WHO MONICA Project MONICA Manual (1998–1999) [Internet]. Geneva: World Health Organization [cited 2011 Aug 23]. Available from: http://www.ktl.fi/publications/monica/manual/index.htm

[35] Wu ZS, Yao CH, Zhao D Epidemiological study about incidence and mortality rate of stroke in Chinese. Chin J Epidemiol. 2003;24:236–9 (in Chinese)

[36] National Bureau of Statistics of China. Report of the sixth National Census in 2010. 1st ed. Beijing: China Statistics Press; 2012.

[37] Center for Health Statistics and Information of Ministry of Health of China. An Analysis Report of National Health Services Survey in China, 2008. 1st ed. Bejing: Peking Union Medical College Press; 2009.

[38] Kitamura A, Sato S, Kiyama M, Imano H, Iso H, Okada T, Trends in the incidence of coronary heart disease and stroke and their risk factors in Japan, 1964 to 2003: the Akita-Osaka study. J Am Coll Cardiol. 2008;52:71–9 10.1016/j.jacc.2008.02.075 18582638

[39] Ca M, Qian JC Trends of smoking prevalence of Chinese women and associated factors. Chin Hosp Stat. 2009;16:289–92 (in Chinese)

[40] Iso H Changes in coronary heart disease risk among Japanese. Circulation. 2008;118:2725–9 10.1161/CIRCULATIONAHA.107.750117 19106396

[41] Yang GH, Ma JM, Liu N, Zhou LN [Smoking and passive smoking in Chinese, 2002]. Zhonghua Liu Xing Bing Xue Za Zhi. 2005;26(2):77–83 15921604

[42] Ma GS, Zhu DH, Hu XQ, Luan DC, Kong LZ, Yang XG The drinking practice of people in China. Ying Yang Xue Bao. 2005;27:362–5 (in Chinese)

[43] Wu YF, Ma GS, Hu YH, Li YP, Li X, Cui ZH, [The current prevalence status of body overweight and obesity in China: data from the China National Nutrition and Health Survey]. Zhonghua Yu Fang Yi Xue Za Zhi. 2005;39(5):316–20 16266540

[44] Wu Y, Zhou B, Tao S, Wu X, Yang J, Li Y, [Prevalence of overweight and obesity in Chinese middle-aged populations: Current status and trend of development]. Zhonghua Liu Xing Bing Xue Za Zhi. 2002;23(1):11–5 12015101

[45] Ministry of Health and Welfare. Annual Report of the National Health and Nutrition Survey in 2009. Tokyo: Daiichi Publishing; 2010.

[46] Flegal KM, Carroll MD, Ogden CL, Curtin LR Prevalence and trends in obesity among US adults, 1999–2008. JAMA. 2010;303:235–41 10.1001/jama.2009.2014 20071471

[47] Chen JC, Gu DF, Wu XG, Duan XF, Gan WQ, Cao TX, Changes of dietary nutrient intakes from 1991 to 1999 among farmers living in Fangshan district of Beijng. Chin J Clin Rehabil. 2005;9:14–6 (in Chinese)

[48] Pan ZQ, Gu DF, Wu XG, Cao TX, Xu XS, Xie BY Nutritional intakes of farmers in Beijing suburb. Chin Circ J. 1995;11:672–703 (in Chinese)

[49] Xie G, Mai J, Zhao L, Liu X [Physical activity status of working time and its change over a ten-year period in Beijing and Guangzhou populations]. Wei Sheng Yan Jiu. 2008;37(1):33–6 18421859

[50] Bell AC, Ge K, Popkin BM Weight gain and its predictors in Chinese adults. Int J Obes Relat Metab Disord. 2001;25:1079–86 10.1038/sj.ijo.0801651 11443510

[51] Du S, Lü B, Wang Z, Zhai F [Transition of dietary pattern in China]. Wei Sheng Yan Jiu. 2001;30(4):221–5 12561521

[52] Gu D, Reynolds K, Wu X, Chen J, Duan X, Muntner P, Prevalence, awareness, treatment, and control of hypertension in china. Hypertension. 2002;40:920–7 10.1161/01.HYP.0000040263.94619.D5 12468580

[53] Guo JP, Huang JY, Cao YF, Yang YJ, Shen FY, Wang Y, Survey of hypertension in a rural area of Shanghai city. Chin Gen Pract. 2007;10:1267–71 (in Chinese)

[54] Gu D, Reynolds K, Duan X, Xin X, Chen J, Wu X, Prevalence of diabetes and impaired fasting glucose in the Chinese adult population: International Collaborative Study of Cardiovascular Disease in Asia (InterASIA). Diabetologia. 2003;46:1190–8 10.1007/s00125-003-1167-8 12879248

[55] Han DL, Feng YS, Liu RT, Chen GX Survey on the suffering status of hypertension and coronary heart disease and cerebral apoplexy among countryside population over 35 in Linyi city. Prev Med Trib. 2007;13:209–10 (in Chinese)

[56] Fang XH Epidemiology of stroke and its influencing factors. Chin J Cerebrovasc Dis. 2004;5:233–7 (in Chinese)

[57] Wu ZS, Yao CH, Zhao D, Wu GX, Wang W, Liu J, Multiprovincial monitoring of the trends and determinants in cardiovascular disease (Sino-MONICA project)-I Morbidity and mortality monitoring. Chin J Cardiol. 1997;25:6–11 (in Chinese)

